# New Appliances and Things Medical

**Published:** 1900-07-28

**Authors:** 


### NEW APPLIANCES AND THINGS MEDICAL.

**[We shall be glad to receive, at our Office, 28 & 29, Southampton Street, Strand, London, W.C., from the manufacturers, specimens of all new preparations and appliances which may be brought out from time to time.]**

### W. R. WARNER'S MEDICINAL PREPARATIONS.

(Sole Agent, F. Newbery and Sons, 27 and 28, Charterhouse Square, London, E.C.)

W. R. Warner and Co. are now supplying very excellent preparations, several of which have been sent us for examination. The "Elixir Salicylic Co." is a specially useful preparation for rheumatism or gout, containing salicylic acid, iodide of potassium, cimicifuga, and tincture of gelsenimum. In addition to the valuable combination of drugs of which the elixir consists, it possesses the further advantage of containing soda in excess, thereby obviating the irritating properties of the acid. The Vichy tablets or lentiforms may be dissolved in plain water, and supply a draught of definite medicinal strength which closely represents the natural spring water. The Lithia tablets contain two grains of citrate of lithia, and when dissolved in water supply an accurate dosage of the drug. The Nervitone tablets, which are elegantly and pleasantly coated, contain $\frac{1}{100}$ of a grain of phosphorus, combined with small doses of asafœtida, sumbul, carbonate of iron, and nux vomica. It is a most happily conceived preparation for neurasthenia and general debility. The effervescing Bromo-soda is a combination of caffein and bromide of soda. When one teaspoonful is dissolved in water it constitutes a most refreshing draught, and is particularly indicated in cases of fatigue, exhaustion, or nerve depression. Ingluvin is a special digestive ferment to be used in those cases which are generally treated by pepsin. It appears to possess properties which are not present in other digestive ferments, and to be particularly indicated in the vomiting of pregnancy.

### CHELTINE FOODS.

(Worth's Food Syndicate, Ltd., Cheltenham.)

Worth's Cheltine foods consist of the following varieties: Diabetic food, Diabetic biscuits, infants' food, "Perfect Invalids" food, dyspeptic food, and anæmic food. The diabetic food is a fine granulated powder, to be prepared for use by mixing two tablespoonfuls with half a pint of cold water and boiling for five minutes. The food prepared in this manner is a complete food, containing a very large proportion of soluble carbohydrates, about one-fifth as much albuminoid matter, and a trace of fat, with a considerable percentage of mineral constituents. Authorities do not appear to be agreed as to the exact proportions of carbohydrates, proteids, and fats indicated during the various stages and degrees of diabetes. Practitioners should, therefore, study for themselves the proportion of these nutritive elements before prescribing any particular variety of prepared food. According to the analysis of Mr. Ellwood, the Cheltene Diabetic Food is as follows: Albuminoids, 14·86 per cent.; carbo-hydrates and cellulose, 76 per cent.; fatty matter, 2·84 per cent. The diabetic biscuits, except in so far that they contain a larger percentage of fat, practically correspond in percentage to the above figures. The remaining foods, although differing to some degree as regards their percentage composition, agree as regards their main features; that is to say, they are foods chiefly consisting of carbohydrates, more or less soluble and diffusible, according to the manner of preparation. They require little assistance from the natural digestive processes of the body before they are in a condition to be absorbed, and when foods of this class are indicated they will be found to answer the purpose admirably.

